# Neuronal representation of visual motion and orientation in the fly medulla

**DOI:** 10.3389/fncir.2012.00072

**Published:** 2012-10-09

**Authors:** Christian Spalthoff, Ralf Gerdes, Rafael Kurtz

**Affiliations:** Department of Neurobiology, Bielefeld UniversityBielefeld, Germany

**Keywords:** vision, invertebrates, orientation selectivity, calcium imaging, motion detection

## Abstract

In insects, the first extraction of motion and direction clues from local brightness modulations is thought to take place in the medulla. However, whether and how these computations are represented in the medulla stills remain widely unknown, because electrical recording of the neurons in the medulla is difficult. As an effort to overcome this difficulty, we employed local electroporation *in vivo* in the medulla of the blowfly (*Calliphora vicina*) to stain small ensembles of neurons with a calcium-sensitive dye. We studied the responses of these neuronal ensembles to spatial and temporal brightness modulations and found selectivity for grating orientation. In contrast, the responses to the two opposite directions of motion of a grating with the same orientation were similar in magnitude, indicating that strong directional selectivity is either not present in the types of neurons covered by our data set, or that direction-selective signals are too closely spaced to be distinguished by our calcium imaging. The calcium responses also showed a bell-shaped dependency on the temporal frequency of drifting gratings, with an optimum higher than that observed in one of the subsequent processing stages, i.e., the lobula plate. Medulla responses were elicited by on- as well as off-stimuli with some spatial heterogeneity in the sensitivity for “on” and “off”, and in the polarity of the responses. Medulla neurons thus show similarities to some established principles of motion and edge detection in the vertebrate visual system.

## Introduction

Even though the visual systems of insects and vertebrates differ in many structural aspects, still they utilize many design principles in common, such as their parallel processing of information about color, form and motion (Sanes and Zipursky, 2010). In vertebrates, some of the neuronal mechanisms that are necessary for pattern discrimination are relatively well known. Prominent examples are the on- and off-channels in the retina and their integration into orientation-selective units in the visual cortex (Ferster and Miller, 2000; Hirsch and Martinez, 2006).

In compound eye insects, these mechanisms are less well studied. Motion detection has been the main focus in several species (Nordstrom and O'Carroll, 2009; Borst et al., 2010). Recently, several studies demonstrated that, similar to the vertebrate visual system, photoreceptor signals are split into separate “on” and “off” channels (Joesch et al., 2010; Reiff et al., 2010; Clark et al., 2011). However, how early in the visual pathway this separation occurs, how strict it is, and how these channels interact in the motion pathway is still controversial (Reiff et al., 2010; Clark et al., 2011; Eichner et al., 2011). Thus, the preliminary steps leading to motion detection remain elusive, and it is unknown whether the extraction of stationary features, such as orientation, interacts with the computation of motion.

The fly visual system consists of the retina and three neuropils, the lamina, the medulla and the lobula complex, which is split into an anterior part (lobula) and a posterior part (lobula plate). The retina contains within each ommatidium six outer photoreceptors, R1–R6, and two central ones, R7 and R8. Whereas R1–R6 terminate in the lamina, R7/R8 bypass the lamina and terminate in the medulla (Meinertzhagen and O'Neil, 1991). These two neuropils contain arrays of retinotopically arranged modules, the lamina cartridges and the medulla columns. Each of these modules receives primarily input from a single retinal sampling point. The most prominent lamina neurons, the large monopolar cells L1 and L2 are morphologically and functionally well characterized (Fischbach and Dittrich, 1989; Takemura et al., 2008). L1 and L2, which receive direct input from R1–R6 via an inhibitory synapse, both respond in a transient fashion to brightness changes. In addition to further columnar cell types (L3–L5 and T1) lateral connections are formed by amacrine and widefield neurons, and synaptic endings of centrifugal elements (C2, C3) are present (Strausfeld and Campos-Ortega, 1977; Fischbach and Dittrich, 1989; Meinertzhagen and O'Neil, 1991; Takemura et al., 2008). Thus, even in the lamina, lateral connectivity and feedback is present, but local signal processing appears to predominate. More extensive lateral interconnectivity arises on the level of the medulla, which contains a network of interneurons crossing the boundaries between columns (Fischbach and Dittrich, 1989). Lateral comparisons of local signals are required for the computation of form and motion information. Thus, the medulla is a good candidate neuropil to extract these visual features from local input and to supply this information to more specialized downstream brain regions. The large lobula plate neurons, which integrate local motion inputs and thus respond in a direction-selective way to motion in a large part of the visual field have been studied extensively (Borst et al., 2010), and some types of neurons of the lobula have been characterized (Gilbert and Strausfeld, 1991; Nordstrom et al., 2006; Okamura and Strausfeld, 2007; Trischler et al., 2007). In contrast, hampered by the small size of the neurons in the medulla, the neural substrates of local motion or shape detection are still enigmatic although detailed accounts of the anatomical structure of the medulla exist for *Drosophila* (Fischbach and Dittrich, 1989) and *Musca* (Strausfeld, 1976).

Extracellular recordings in the medulla have yielded a first documentation of neurons which respond selectively to the orientation of moving or stationary gratings (Bishop et al., 1968). Later, distinct types of orientation-selective and direction-selective neurons in the medulla were identified by intracellular recording combined with dye staining of individual cells (Gilbert et al., 1991; Douglass and Strausfeld, 1998, 2003). These experiments, however, were only successful in brief recordings from a single neuron of each type, still leaving open the question whether orientation selectivity or motion sensitivity is ubiquitously represented across various cell types in the medulla.

We addressed these issues by examining the responses of medulla neurons through population staining with calcium sensitive dyes, circumventing the need for intracellular recordings or genetically induced labeling methods. Our results point to the fact that, once more, surprisingly similar functional design principles are realized in vertebrate and invertebrate visual systems.

## Materials and methods

### Flies

Blowflies (*Calliphora vicina*) were raised in the department's stock at 25°C in a 12 h light/12 h dark cycle. Experiments were carried out on females collected <3 days after eclosion.

### Preparation

**Figure 1:**
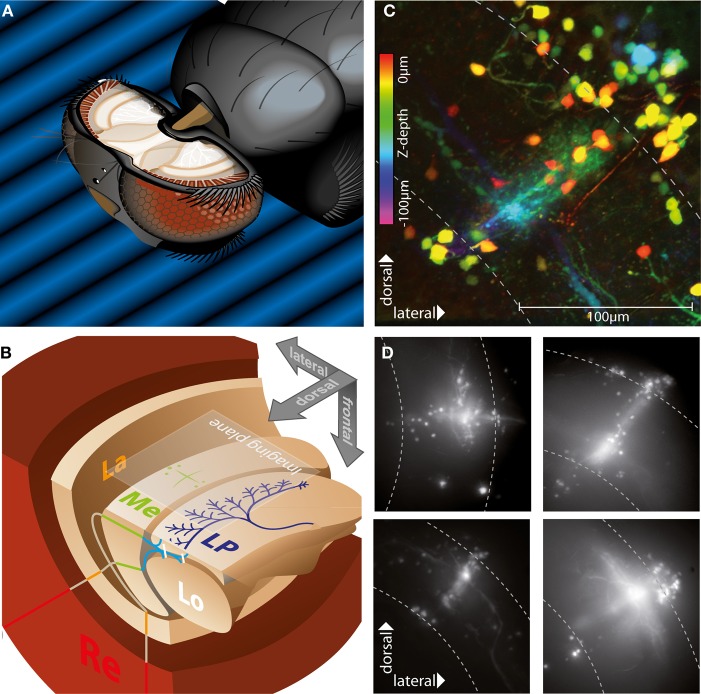
**Morphology of stained neurons in the medulla**. **(A)** Positioning and preparation of the fly. The head of the animal is tilted forward and fixed with beeswax to look down at a screen presenting the visual stimulus. The caudal head capsule is cut open to access the optic lobes, so that our plane of imaging corresponded to the caudal brain surface and the plane in which the stimuli were presented. **(B)** Schematic of visual neuropils of the right hemisphere, same alignment as in a, showing retina (Re), lamina (La), medulla (Me), lobula (Lo), and lobula plate (LP). Two planes represent a horizontal (lower left) and frontal (upper quadrant) cut through the brain. A large cell of the lobula plate (dark blue) and an exemplary staining pattern in the medulla (green) are drawn in for clarity. The two lines represent the propagation of retinotopic information through the system, with inputs from the frontal and lateral visual fields crossing at the first optic chiasm between lamina and medulla and at the second chiasm between medulla, lobula and lobula plate. The imaging plane of the camera is aligned with the caudal surface of the brain, so that dorsal is on top. **(C)** Collapsed confocal image stack of an exemplary Calcium Green dextran population staining in the medulla, showing a columnar bundle of axons, perpendicular tangential dendrites, and two groups of somas located superficially near the proximal and distal boundaries of the medulla. Color coding of the structures indicates depth. Medulla boundaries are indicated by the dashed lines. **(D)** Collapsed image stacks obtained with widefield microscopy of four examples of population staining in different parts of the medulla. Stainings usually show two groups of somas and tangential as well as columnar structures, with the crossing point corresponding to the injection site in different layers of the medulla.

After affixing the fly's thorax in a horizontal position to a glass cover slide, legs, antennae, proboscis and the digestive tract were removed and the openings in the cuticle closed with beeswax. The head was pulled downwards and attached to the thorax so that the caudal surface of the head was aligned horizontally parallel to the glass slide (Figure 1A). An opening was cut into the right half of the caudal head cuticle and the dorsal thorax was opened to insert the reference electrode. We superfused the exposed tissue with insect Ringer solution (Kurtz et al., 2006) to prevent desiccation during the staining, and later to provide an immersion medium for microscopy.

### Calcium dye loading

We pulled micropipettes (Warner G150TF-4 glass tubing, Warner Instruments, Hamden, CT, USA) with a Sutter P-97 puller (Sutter Instruments, San Rafael, CA, USA) to have resistances of 8–10 MΩ and filled them with 5% Calcium Green-1 dextran (3000 MW, Molecular Probes, Eugene, OR, USA) in 50 mM HEPES/5 mM KCl in the tip and 1 M KCl in the back end. Under visual control through a stereo microscope, we inserted the micro-electrode superficially into the medulla and applied negative current pulses (8 μA amplitude, 30 ms pulses with 270 ms intervals) for 45 s with a high-gain micro-electrode amplifier (VF-1800, Bio-Logic, Claix, France) to label the neurons by electroporation (method modified after Fujiwara et al., 2009). In some animals, a second and third injection site was stained in the same manner. Flies were left to incubate for 1–3 h at room temperature.

### Morphological examination and calcium imaging

We recorded relative cytosolic Ca^2+^ concentration changes by epifluorescence imaging of Oregon Green dextran emission using a water immersion 40× (Olympus LUMPlan FI/IR 40×/0.8 W) or 25× (Leica HCX IRAPO L 25×/0.9 W) objective at an upright fixed-stage microscope (Leica DMLFSA) equipped with an electron-multiplying charged-coupled device (EMCCD) camera (Andor iXon DV887-BI, Andor Technology, Belfast, Northern Ireland). Image resolution was 512 × 512 pixels at frame rates of 15–30 Hz. 470 nm excitation light was provided by a Leica Fluo LED 4000 light source (filter set: excitation BP 470/40 nm, dichroic mirror 510 nm, emission LP 515 nm and BP 530/50 nm). In some of the preparations the morphology of the dye-stained neurons was inspected with a confocal microscope (Leica TCS SP2, equipped with a water-immersion objective HCX APO L UV-I 40×/0.80).

### Visual stimulus

**Figure 2:**
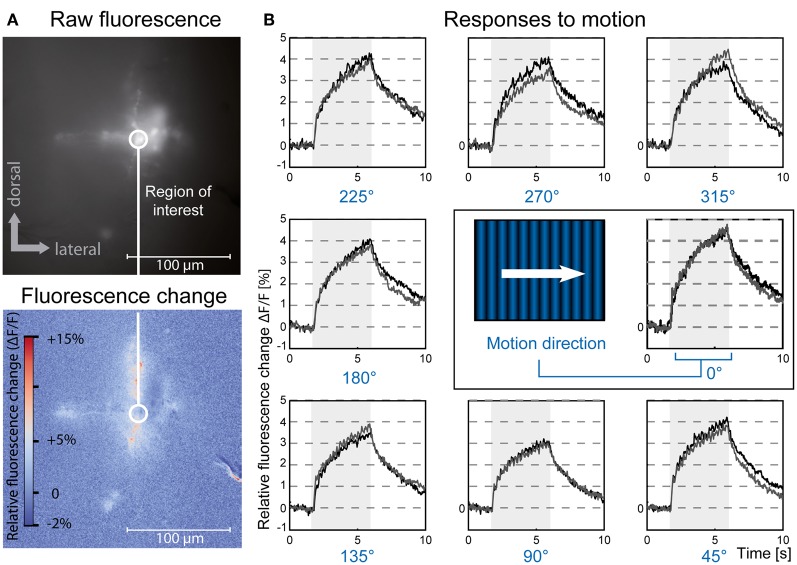
**Optical imaging of medulla neurons during presentation of moving gratings**. **(A)** Top: 10-frame average image of a population of medulla neurons filled with Calcium Green dextran. Bottom: 10-frame average image showing relative differences in local fluorescence intensity during the last 300 ms of stimulation compared to baseline fluorescence. The circle outlines a typical region of interest centered on the injection site. The branch-like structure visible in the bottom right is an artifact caused by movement of a superficial trachea. **(B)** Relative fluorescence changes (Δ*F*/*F*) in the region of interest indicated in **(A)** in response to a grating drifting in eight directions, two repetitions (gray and black traces). Gray rectangles indicate duration of stimulus movement. Stimuli were presented on a 60 Hz TFT screen. The data presented here is the same as used for Figure 4A.

**Figure 3:**
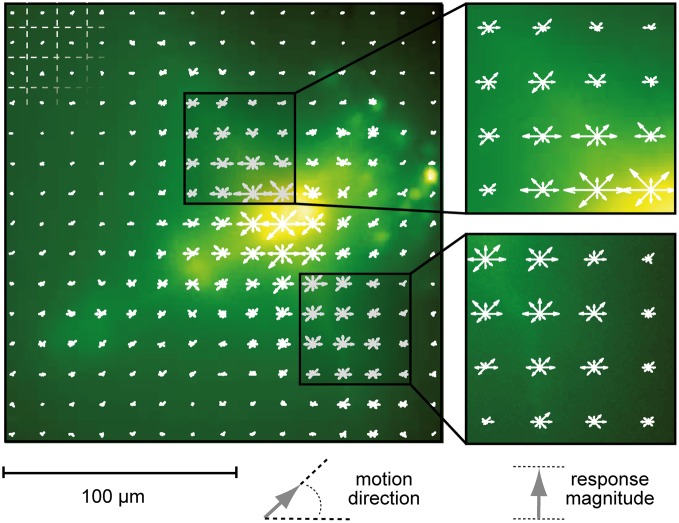
**Localization of motion responses**. Local changes in Calcium Green fluorescence induced by stimulation with motion in eight directions. The length of each arrow represents average fluorescence increase (Δ*F*) in the underlying square region of the image (indicated by the grid in the upper left corner of the image). Motion direction is depicted by the direction of the arrow. Fluorescence signals were time-averaged over the entire period of motion stimulation. Stimuli were presented on a 60 Hz TFT screen.

**Figure 4:**
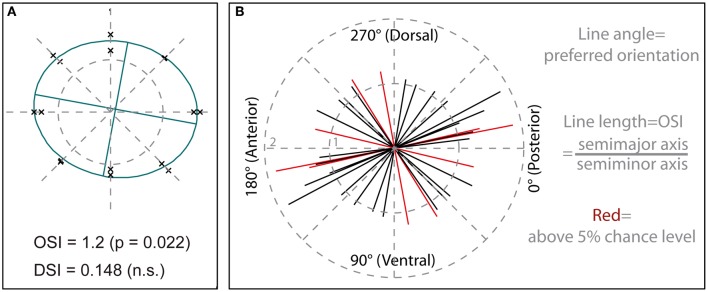
**Quantification of orientation selectivity**. **(A)** Calcium responses within a central region of interest (see Figure 2A), time-averaged over the entire period of motion stimulation, are depicted in a polar plot. Each data point represents a single recording, with distance from the center corresponding to mean response amplitude and position relative to the center of the plot corresponding to stimulus direction. Data from the same staining as shown in Figure 2. The ellipse plotted in green represents a least- square fit to the data, with the ratio of semimajor to semiminor axis giving the orientation selectivity index (OSI) and the ratio of center displacement to semimajor axis the direction selectivity index (DSI). The OSI yields a value of 1 for recordings that are not orientation selective and would, theoretically, rise to infinity when responses are obtained only for a single orientation of the grating. The DSI can vary between 0, indicating no directional selectivity, and 1, indicating maximum directional selectivity. **(B)** Preferred orientations and strengths of orientation selectivity, indicated by line length, of medulla cells. Each line (*N* = 18) represents averaged signals from one population staining. Lines colored in red show significant orientation selectivity (*P* < 0.05). Stimuli were presented on a 60 Hz TFT screen.

**Figure 5:**
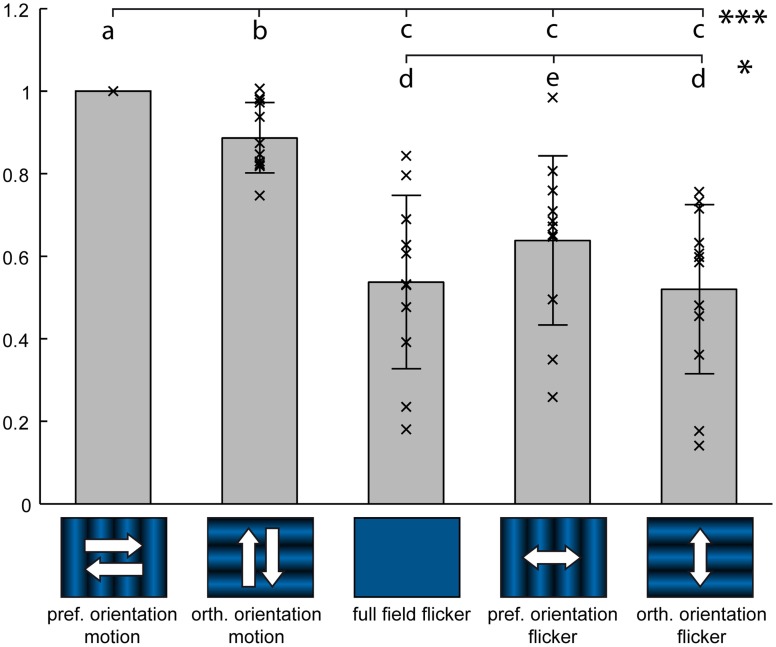
**Flicker stimuli elicit weaker responses than motion**. Response of 12 medulla cell populations during stimulation with two orthogonal orientations of motion, full-field flicker, and two corresponding orientations of counter-phase flicker. Each data point represents average responses from one staining and 2–3 trials, from a circular Region of Interest centered on the injection site. Data has been normalized to preferred orientation motion response. Gray bars represent the mean ± standard deviation. Different letters denote significant difference (Wilcoxon signed-rank test) at *P* < 0.001 for letters a–c or *P* < 0.05 for letters d,e. Stimuli were presented on a 60 Hz TFT screen.

**Figure 6:**
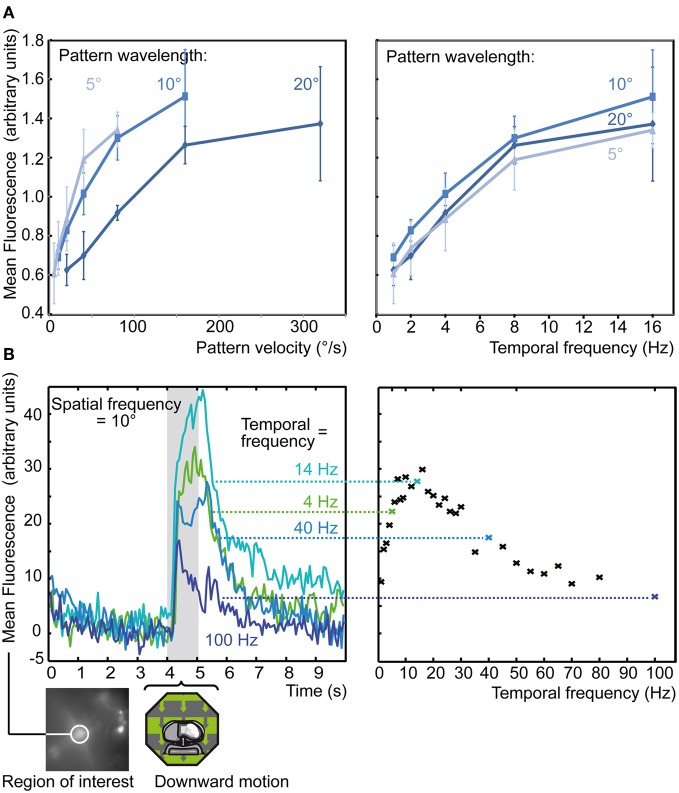
**Tuning to different temporal and spatial frequencies**. **(A)** Average response of medulla population stainings to 4 s motion of a drifting sine-wave grating with preferred orientation at different velocities (left) or temporal frequencies (right) and pattern wavelengths. Each series was normalized to the average response to the set of all 15 stimuli. Each data point represents normalized average response from 6 stainings and 19 single recordings, each from a circular ROI each centered on the injection site, ±SEM. Stimuli were presented on a 60 Hz TFT screen. **(B)** Response of a single population staining (bottom left) to 1 s of downward motion with varying temporal frequency. Each data point represents a single trial response, from a circular ROI centered on the injection site, with four corresponding example Δ*F* traces shown to the left. Stimuli were presented on a high-speed LED array.

**Figure 7:**
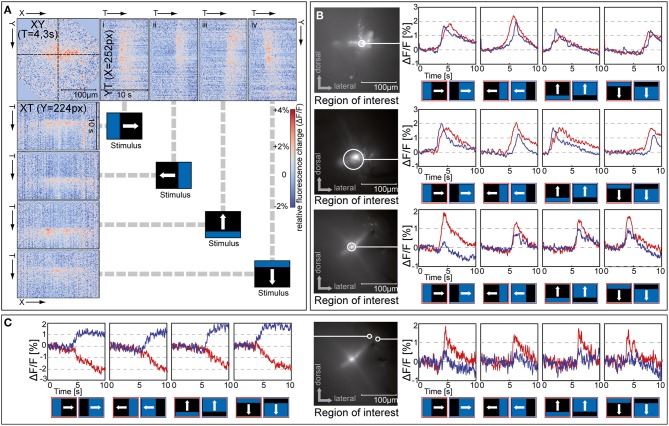
**Responses to moving bright and dark edges**. **(A)** Orthogonal views of an x-y-t-stack of relative fluorescence changes (Δ*F*/*F*) in a staining of the distal medulla (same cell as in C, image rotated by 20°). The top left plot shows the spatial activation pattern of the cell population at a point in time, while the plots at the top (i–iv) and to the left show the temporal activation pattern of a single row or column in response to a bright moving bar stimulus. Similar results were obtained in five further stainings. **(B,C)** Relative fluorescence changes (Δ*F*/*F*) in the region of interest indicated in response to a bright edge (red) and dark edge (blue) drifting in four directions. Regions of interest in *C* are centered on two somas near the distal rim of the medulla. Stimuli were presented on a 60 Hz TFT screen.

We used two different stimulus devices to account for the shortcomings of each method: first, a small TFT display was used as a device that allows high flexibility in the design of different visual stimuli and that can easily be positioned in the limited space of our microscope setup (data shown in Figures 2, 3, 4, 5, 6A, 7). Second, an LED-based stimulus display was used to allow presentation of visual stimuli at a high frame rate (data shown in Figure 6B and data mentioned in the last paragraph of the results section “Neuronal activity is tuned to grating orientation, but not motion direction”). The TFT display (F510EK005, Reikotronic, Cologne, Germany, 10.4″ LED backlit LCD, nominal maximal white luminance: 1000 cdm^−2^) with a frame rate of 60 Hz was used to present various motion or flicker stimuli. Response to sine-wave gratings drifting in one out of eight tested directions (temporal frequency: 1–16 Hz, spatial frequency: 5, 10, or 20°), were compared with the responses to the corresponding counter-phase flicker stimuli, which were equivalent to the motion stimuli in their spatial and temporal pattern properties and their orientation. Additionally, full field flicker stimuli with the same temporal frequencies were used. The screen covered ca. 40° in elevation and 50° in azimuth to both sides from a point centered in front of the fly. Stimuli were designed using OpenGL/Vision Egg (Straw, 2008) and presented using the blue channel. The light from the TFT display was filtered by a dichroic glass filter (Blueberry 8, SP 515 nm, Lee Filters, Hampshire, GB) which prevented bleed-through of the stimulus light to the fluorescence signal. The brightness values (Minolta spot luminance meter LS-100) of the stimuli were 11 cdm^−2^ for the brightest pattern regions and 0.2 cdm^−2^ for the darkest pattern regions. Thus, the Michelson contrast of the sine-wave grating was 0.96. We note that the effective contrast seen by the fly is presumably somewhat lower, because fluorescence excitation light penetrates the fly's head and causes steady illumination of the photoreceptor layer. Resulting from the high temporal resolution of the fly's visual system, the use of stimulus devices with a refresh rate below 150 Hz might cause coupling of neuronal activity to the refresh rate. Although these issues are not likely to be critical for the topic of the present study, we validated our major conclusions by the use of an LED-based stimulus display.

For these experiments we used a board of 22 × 45 green LEDs (each ~4.8 × 2.5 mm, emission maximum at ~570 nm, covered with a LP 550 nm filter to reduce cross talk with fluorescence emission light) to simulate a moving high contrast square wave pattern (temporal frequency: up to 200 Hz, spatial frequency: 10°, mean luminance through filter of bright/dark stripes: 52/0.4 cdm^−2^, resulting Michelson contrast: 0.98). The LED board was updated at a rate of 4 kHz using an analog-to-digital converter (DT2801A, Data Translation, Marlboro, MA, USA) and program routines written in C (Borland, Scotts Valley, CA, USA). The visible pattern consisted of an octagonal area centered in the frontal visual field with an angular extent of ~80 × 80°. The LED board could be pivoted about the center, allowing changes of motion direction in 45°-steps while leaving the visible area constant. The stimulus consisted of 2 s presentation of the stationary pattern, followed by 4 s of motion or flicker (1 s for data presented in Figure 6B), and 4 s stationary pattern again. Stimulus presentation order was pseudorandom, and stimulus presentation was paused for at least 10 s before starting the next recording.

### Data analysis

Camera control and image acquisition were performed using ImSpector 3.20 (LaVision Biotec, Bielefeld, Germany). We used Matlab (The Mathworks, Natick, MA, USA) and ImageJ (US National Institutes of Health, Bethesda, MD, USA) for data analysis. Ca^2+^ concentration signals were evaluated as background-subtracted pixel-wise changes from baseline levels of the fluorescence of the Ca^2+^-sensitive dye divided by the baseline value (Δ*F*/*F*_0_). For the baseline values (*F*_0_) we used the average of the images during the first 1.5 s in the series. For presentation as mean Δ*F* false-color-plots, the image stacks were filtered with a 2-pixel half width xy-gaussian blur and a 2-frame running average in *t*-direction.

For calculation of orientation and direction selectivity indices (OSI and DSI), we fitted a conic ellipse to a polar plot of the responses at different motion directions using a linear least square criterion (modified from Matlab function “fit_ellipse”; Gal, 2003). After transformation to standard ellipse form, this gives the 5 parameters semimajor axis a, semiminor axis b, orientation ϕ and center coordinates x and y. We define the orientation selectivity index as OSI = *a*/*b*, and the direction selectivity index as DSI = (*x*^2^ + *y*^2^)^0.5^/*a*.

## Results

### *In vivo* local electroporation stains columnar and tangential structures in the medulla

Loading of neurons with Calcium-Green dextran via electroporation to injection sites near the surface of the medulla (Figures 1A,B) led to stereotypic “cross-shaped” staining patterns (Figure 1C). Visible structures consisted of somas mostly located superficially above the distal and proximal boundaries of the medulla, bundles of straight axonal projections crossing the medulla in a radial direction, and tangential structures, i.e., neuronal elements arranged orthogonally to these bundles (Figure 1D). The point of intersection of these columnar and tangential structures was centered on the injection site and was in different stainings located in different medulla layers. Based on the number of somas visible, the total number of cells stained in one injection was usually between 20 and 40. Anatomical studies imply an overall number of 60–100 cells within a single column of the medulla (Strausfeld, 1976; Fischbach and Dittrich, 1989).

Although an unequivocal identification of the cells stained by local electroporation was not possible an attempt to classify the types of neurons can be made based on their similarity with neuronal morphology characterized in anatomical studies. The columnar elements usually showed a high number of axons located in the medulla, diffuse arborizations in proximal and distal layers, and relatively small cell bodies located above the distal rim of the medulla, a structure which is similar to the anatomy of Mi (“Medulla intrinsic”) neurons described in Drosophila (Fischbach and Dittrich, 1989). A smaller number of cells had axonal protrusions which extended across the proximal rim of the medulla and dived down into the gap between medulla and lobula plate, a structure described for TM (“trans-medulla”) neurons, which possess distally located somas, as well as T2 cells (Fischbach and Dittrich, 1989; Douglass and Strausfeld, 2003), which have their somas located near the surface of the proximal medulla.

The tangential structures had comparatively thick processes, which in cells located more superficially were mostly smooth, but in deeper structures often studded with distinct varicosities. Depending on the injection site these neurons were located in different layers, and their arborizations were mainly confined to a single depth level, running parallel to the caudal surface of the medulla. Such a ramification pattern is found in some of the Mt (“Medulla tangential”) neurons as well as in amacrine cells (Fischbach and Dittrich, 1989).

No stained structures extending into the lamina were visible, which would correspond to L1–L5 type lamina interneurons or R7 or R8 photoreceptors, but a co-staining of these cells cannot be ruled out, because the curvature of the first optic chiasm obstructs the view to these cells from a caudal observation point.

### Neuronal activity is tuned to grating orientation, but not motion direction

To analyze whether selectivity for motion direction or pattern orientation is represented in the responses of neurons of the fly medulla we presented drifting sine wave gratings in the frontal visual field. These stimuli evoked robust and wide-spread increases in cytosolic calcium level in tangential as well as columnar structures of the medulla (Figure 2). We compared Δ*F*/*F* responses in a given region of interest for four different pattern orientations and two directions each (Figure 2B). Regions of interest for data evaluation were centered on the crossing between tangential and columnar structures, since signals usually were strongest in this area. While other regions of the cells often showed faster or slower time courses of the signal, differences in the directional tuning of different regions in a single staining were not prominent. In the example shown in Figure 3 a preference for horizontal motion (i.e., for the grating with vertically oriented stripes) was present in regions along the medulla column as well as in the tangential structures stretching out to both sides of the column. Regardless of the location, responses to the two opposing directions of motion of a grating with the same orientation were similarly strong.

To quantify the selectivity to stimulus orientation and directional selectivity for each staining, we took the calcium responses within a central region of interest (similar to the one shown in Figure 2A), time-averaged over the entire period of motion stimulation, and plotted each recording as a data point in a polar plot (Figure 4A). In this plot the distance of a data point from the center represents the response magnitude during motion in the direction indicated by the position of the data point. We least-square fitted a standard ellipse to these points (Batschelet, 1981) and took the major axis orientation θ as the preferred orientation. Note that this definition of preferred orientation denotes an axis of motion, which is orthogonal to the spatial orientation of the grating pattern. As a measure for orientation selectivity we took the ratio between the semimajor and the semiminor axis of the ellipse, called orientation selectivity index (OSI) in the following. The strength of directional selectivity was quantified by taking the displacement of the center of the ellipse from the center of the coordinate system and dividing this value by the semimajor axis of the ellipse to normalize for differences in ellipse size, called DSI in the following.

To test for statistical significance, we used a Monte-Carlo-approach to estimate the distribution of chance level OSI and DSI values obtained for the data set of a given recording. As a basic principle of this approach, the recorded data traces were randomly assigned to the different stimulus conditions. Standard ellipses were fitted to each of 10,000 control datasets generated by this random shuffling procedure. As a measure of error probability we then determined how many of the fits to these random datasets produced OSI or DSI values higher or equal to the measured values.

Results for 18 stainings are shown in Figure 4B. Preferred orientations are slightly biased to the horizontal axis, corresponding to movement in the anterior–posterior or posterior–anterior direction, with 12 of 18 measurements falling into a range of ±45° around the horizontal. Five of the stainings showed orientation selectivity above 5% chance level, with OSIs reaching maximum values slightly below 2.

DSIs were between 0.02 and 0.20, and never showed statistical significance above chance level. Selectivity for grating orientation and motion direction was also tested using an LED board, which allowed us to present stimuli at a higher refresh rate than with the TFT display (4 kHz vs. 60 Hz). Four out of nine stainings in this series of experiments showed significant orientation selectivity as tested by the Monte-Carlo-approach (*p* < 0.05, data not shown). One out of the four orientation-selective stainings also showed directional selectivity (*p* < 0.05).

These results suggest that directional selectivity is not yet or only sporadically present on the processing stage of the medulla columns, but that the orientation of moving stimuli is represented in the signals of medulla neurons. However, since our recordings always consist of a population-average of the stained medulla cells, we cannot rule out that individual signals with stronger selectivity for orientation or direction are pooled into an average with a broader tuning.

### Responses to flicker are weaker than to motion

Motion stimuli always induce local modulations of brightness, which might also elicit responses in cells that are not selective for motion. We tested whether medulla elements respond stronger to motion than to brightness modulations that lack the motion-defining spatio-temporal correlations. For this, we stimulated the cells with several versions of flicker inducing temporal brightness modulations similar to the motion stimuli shown in Figure 2B.

Two orientations of a sine wave grating, which smoothly inverted their phase with a 4-Hz-frequency (counter-phase flicker), were tested. Additionally, a 4-Hz sinusoidal untextured brightness modulation, which lacks pattern orientation information (full-field flicker), was used. A direct comparison of stationary counter-phase flicker stimuli with moving gratings is problematic because, apart from the minima and the maxima of the sinusoidal pattern, counter-phase flicker induces a lower local brightness modulation than moving gratings and full-field flicker. For neurons with receptive fields much smaller than the pattern wavelength, this difference in local brightness modulation between counter-phase flicker and motion can be compensated by shifting the flicker grating to align one of its minima or maxima with the receptive field. Therefore, we varied the position of the flicker grating in four steps, each equal to 1/8 of the pattern period.

We found that responses to counter-phase flicker at any of the tested phase positions, as well as full-field flicker, were significantly lower than to motion stimuli of the same temporal frequency (Figure 5). Orientation preference for counter-phase flicker was the same as for motion. While the lowered contrast of the counter-phase stimuli in comparison with the moving grating makes a quantitative comparison of the responses difficult, the attenuated response to full-field flicker in comparison to motion corroborates the presence of spatial filtering, resulting in orientation preference, on the level of the medulla.

### Medulla neurons are tuned to temporal frequency

Recordings from the direction-selective lobula-plate tangential cells (LPTCs) of the fly (Egelhaaf et al., 2002; Borst et al., 2010) usually show responses that depend not only on the velocity of a stimulus, but also on its spatial structure (Hausen, 1982). When drifting gratings with different wavelengths are used for stimulation, the optimum velocity is shifted, such that the temporal frequency remains at a fixed value. As fixed tuning to temporal frequency (rather than velocity) is one of the key predictions of a correlation-type motion detector, the Hassenstein-Reichardt model (Hassenstein and Reichardt, 1956), this property of LPTCs has been taken as evidence that this type of motion detector is implemented in the input. Temporal frequency optima of LPTCs in *Calliphora* have been estimated at 2–5 Hz during steady state and 10–20 Hz briefly after stimulus onset (Hausen, 1982; Hengstenberg, 1982). To measure the velocity tuning of medulla cells, we used sine wave gratings drifting in a direction that elicits a strong response. Pattern wavelength and temporal frequency of the stimulus were varied to give 15 combinations between 5 and 20° and 1–16 Hz (Figure 6A). All individual stainings show response peaks at temporal frequencies equal to or greater than 8 Hz (right panel), with no difference in response peak visible for the different spatial frequencies. In contrast, when plotting response amplitude versus pattern velocity, different response maxima for each pattern wavelength are obtained (left panel, same data as in the right). Thus, medulla cells show the same strong dependence on temporal frequency as LPTCs, but are tuned to higher frequencies. This conclusion can be made even though the time constants of Calcium Green, which are as for genetically expressed indicators in the range of several hundreds of milliseconds (Hendel et al., 2008), lead to distortions of fast concentration dynamics. First, what is reported by calcium imaging is not so much the fast fluctuation of the calcium current but the gradual increase in cytosolic calcium concentration, which results from the fact that calcium clearance is usually slower than calcium influx (Sala and Hernandez-Cruz, 1990; Kurtz, 2004). Second, if calcium signals at high temporal frequencies were underestimated, the true difference between LPTCs and medulla neurons would even be larger than indicated by our measurements.

Our data suggest that the peak response of medulla neurons might lie even beyond our highest measured stimulus frequency (16 Hz). Since the 60 Hz frame rate of the TFT screen makes presentation of stimuli with higher temporal frequency problematic, we used an LED array to present temporal frequencies of 1–100 Hz (Figure 6B). The responses under these conditions peak at about 15 Hz, which is consistent with the results from the TFT stimulus, and show a marked decrease at temporal frequencies above 20 Hz. This differs from the dynamic properties of lamina monopolar cells, the neurons forming the major input pathways to the medulla, which respond with high-gain to brightness fluctuations up to more than 100 Hz (Juusola et al., 1995). This comparison suggests that the signals from the lamina are subject to temporal processing in the medulla, such as low-pass filtering, which attenuates the responses to high temporal frequencies. We also tested the temporal frequency tuning using counter-phase flicker presented on the LED array (data recorded in another staining, not shown). Similar to the optimum during motion stimulation, the largest responses to flicker were obtained at about 30 Hz and only weak responses were elicited by frequencies above 100 Hz.

### Tangential structures show retinotopic dendritic input and respond to on- and off-edges

Recently, the demonstration of separate on- and off-channels in the visual pathway of the fly, a common feature in vertebrate vision, has received much attention (Joesch et al., 2010; Reiff et al., 2010; Clark et al., 2011). To examine the representation of “on” and “off” in the medulla, and to characterize the visual field of the stained neurons, we stimulated the fly with bright and dark edges moving across the screen at about 10°/s (Figure 7). We tested five different stainings, two with the injection site located in the distal part of the medulla and three with the injection site located more proximately in the medulla. In all stainings neurons responded to bright as well as dark edges and to motion in all four directions with transient increases in calcium level in tangential as well as in columnar structures (example response to an on-stimulus shown in Figure 7A). From the time during which the calcium signal increased, we estimate the response of this cell population to cover about 1 s of stimulus travel time. This corresponds to a receptive field of about 8°, which is considerably larger than the typical interommatidial distance of about 1.5° in *Calliphora* (Petrowitz et al., 2000). Interactions among neighboring retinotopic columns are a prerequisite for the computation of direction (as well as orientation) selectivity, and interactions spanning as much as four ommatidia in a row have been shown to play a role in fly local motion computation (Schuling et al., 1989).

In response to edges moving in vertical directions, all stainings showed a consecutive localized activation of the tangential structures, which corresponded to the motion of the stimulus through the visual field. This can be seen in the pattern of activation being tilted rightward in the YT-plot for upward motion [Figure 7A(iii)], and leftward for downward motion [Figure 7A(iv)]. Such a localized activation pattern was not present for edges moving horizontally.

In recordings from distal tangential structures, but not in proximal ones, the on- and off-responses differed in their timing. In the examples shown in Figure 7B, top and middle, off-responses showed a clear displacement in peak timing relative to the on-responses, with off-responses leading during progressive and upward motion, and on-responses leading during regressive and downward motion. This suggests input from spatially separated on- and off-channels to the dendrite of the tangential elements located in the distal medulla. In the stainings where stratifications in the proximal layers of the medulla were stained (example in Figure 7B, bottom), such direction-specific differences in response timing were not visible.

Typically, we could observe little separated localization of signals associated with on- and off-stimuli. The same areas within the stained cell population usually responded to all stimuli tested, although in some cases on and off responses appeared to differ in strength (see Figure 7B, bottom). In some stainings, however, some of the somas located at the distal rim of the medulla showed responses that differed from the typical population signal (Figure 7C). One soma showed a strong selectivity for stimulus polarity, responding with sustained increases in calcium to off-stimuli and with sustained decreases to on-stimuli, while a different soma displayed transient signals and a strong preference for on-stimuli. An on/off segregation was previously shown to be present in the fly visual pathway, because blocking one of the major types of output neurons from the lamina to the medulla, L1 or L2, led to selective loss of on and off responses, respectively (Joesch et al., 2010). However, since synaptic terminals of L1 and L2 in the medulla were recently found to respond to on as well as off stimuli (Clark et al., 2011), it was until now unclear whether on/off segregation takes place in a postsynaptic stage of the medulla or later in the lobula complex. The heterogeneity in response characteristics of medulla neurons demonstrated in the present study (Figure 7C) supports the notion that the segregation into separate channels for on and off stimuli takes place in the medulla.

## Discussion

In the medulla of insects visual information is processed by cells that confine their processes into distinct sublayers of the neuropil in a similar fashion as cells of the plexiform layers in the vertebrate retina, suggesting a functional similarity of both systems in the extraction of visual features from the purely retinotopic image represented on the retina (Sanes and Zipursky, 2010). With its large number of densely interwoven neurites and the difficulty of intracellular recordings, still relatively little is known about the representation of visual features in the medulla.

In the present study, we used for the first time local electroporation to stain neurons in the fly visual system with a calcium dye. Our method allowed us to gain access to signals from medulla cell populations and to examine their responses to various spatial and temporal stimulus patterns. Similar to staining with membrane-permeant calcium dyes widely used in vertebrate cortical tissue, identification of individual types of neurons is hampered by the fairly large number of neurons stained in each experiment when using local electroporation. Unfortunately, our attempts to stain smaller numbers of cells were not successful. The use of electrodes with higher resistance as well as the application of current pulses with smaller amplitude led to a prominent decline in the success rate, but not to noticeably more restricted staining patterns. One possibility to characterize single-cell properties is the quantification of somatic calcium signals, as is often done in vertebrate preparations. In our preparation somatic calcium signals were often too weak to be clearly discerned from background fluorescence changes (but see Figure 7C for an exception). A likely reason for the low magnitude of somatic calcium signals is the long distance of the soma from dendritic and axonal structures, which is typical for the morphology of many insect neurons. Additionally, the surface-to-volume ratio of a soma is much lower than that of a small neurite. This difference might result into weaker cytosolic calcium concentration changes of the somas compared to neurites even if membrane calcium currents were similar. In spite of these problems to characterize single-cell properties following electroporation stainings it is possible to outline which functional neuronal features exist in general in the fly medulla.

While studies exist about the processing of color information in the medulla (Morante and Desplan, 2008), it is less clear how motion information is processed and represented in this neuropil, and whether orientation selectivity plays a role as a separate or preliminary computation step to the full directionality emerging at later stages of the visual pathway (Single et al., 1997; Dyhr and Higgins, 2010). Successful electrical recordings and dye stainings are rare in the insect medulla. Directionally selective neurons were characterized in the medulla of the locust, *Locusta migratoria* (Osorio, 1986). In flies, orientation selectivity of single neurons in the medulla was reported in the fleshfly *Sarcophaga bullata* for an amacrine cell, which responded to different orientations of a moving grating with membrane potential oscillations of different amplitudes (Gilbert et al., 1991). By a similar measure, the columnar T1a neuron was in the same study reported to respond slightly direction selective to motion. Later studies on the medulla of blowflies led to the conclusion that the T2 neuron responds selectively to grating orientation, and that strong directional selectivity is present in one type of Y-cell, Y18, whereas another Y-cell, Y19, and the T4 neuron are weakly directionally selective (Douglass and Strausfeld, 1996, 1998, 2003). T4 and T5 were recently shown to be necessary for the computation of directional selectivity, because direction-selective responses of LPTCs were abolished by genetic blockage of these types of neuron in *Drosophila* (Schnell et al., 2012). However, whether or not T4 and T5 themselves are selective for motion direction or pattern orientation cannot be resolved based on this result. Our findings show that orientation-selective responses are a widespread feature of neurons in different layers of the medulla. The concept of non-directional orientation tuning plays an important role in feature extraction across different animal species. Orientation selectivity has been found in neurons of vertebrates like cats (Hubel and Wiesel, 1962), monkeys (Schiller et al., 1976), or birds (Pettigrew and Konishi, 1976), as well as in advanced processing stages in insects (O'Carroll, 1993; Yang and Maddess, 1997; Okamura and Strausfeld, 2007), but their direct input elements remained elusive until now. Due to the fact that we mainly measured population, not single-cell responses, the orientation selectivity values we found provide the lower estimate of the orientation selectivity actually present in the medulla, with single-cell orientation selectivity possibly higher than the values measured. Our findings suggest that information about edge orientation provided by the medulla plays an important role in the downstream processing stages of the insect visual system.

In general, a direct quantitative comparison of neuronal responses to motion and to flicker is difficult because the two types of stimuli differ either in the contrast across space (with full-field flicker) or in the average magnitude of local brightness modulations (with counter-phase flicker). Nevertheless, our finding that the neuronal responses in the medulla to both types of flicker are consistently lower than to motion hints at the possibility of non-directional motion sensitivity. This type of computation has been suggested to occur in the insect visual pathway as a prior step of the directional motion computation performed by the Hassenstein–Reichard-detector (Dyhr and Higgins, 2010), and might serve as an explanation for certain response characteristics of bees in behavioral experiments (Srinivasan et al., 1993). These non-directional behavioral responses show a broad frequency tuning with strong responses to frequencies of 50 Hz and more, while directional responses of fly LPTCs exhibit a much lower temporal frequency tuning that already drops off at 10 Hz. These differences led to the conclusion that non-directional motion computation would have to happen at earlier stages of visual processing (Higgins et al., 2004; Dyhr and Higgins, 2010), with some of this high-frequency information being lost at the later computational stages. This concept is supported by our finding that medulla cells exhibit responses to a broader spectrum of higher temporal frequencies than LPTCs, hinting at a subsequent low-pass-filtering of signals in the computation of directional motion signals.

Several earlier works exist which used flicker to assess the combination of spatial and temporal filtering that forms the basis of motion detection (Borst and Egelhaaf, 1989). Similar to what we found in the medulla, for LPTCs the preferred orientation of a grating in counter-phase flicker was orthogonal to the preferred motion direction, i.e., vertical stripes elicited the largest responses in a cell that responded selectively to horizontal motion (Srinivasan and Dvorak, 1980). This orientation selectivity during flicker stimulation was attributed to the input from an array of sampling units with an excitatory center and two horizontally aligned inhibitory regions, which were called “sustaining units” in recordings from the chiasm between medulla and lamina of the blowfly *Lucilia* (Arnett, 1972). The receptive fields of these units comprised several interommatidial angles (Petrowitz et al., 2000), reaching 6–8° in lateral displacement. A spatial separation of units responding to on- and off-signals was found by Arnett (1972), which bears a striking resemblance to the spatial displacement between on- and off-signals we found in the tangential processes in the distal medulla.

The analysis of the significance of lamina interneurons L1 and L2 to convey information about on- and off-stimuli to the later processing stages has recently made large progress due to the application of genetic tools in *Drosophila* (Rister et al., 2007; Joesch et al., 2010; Reiff et al., 2010; Clark et al., 2011). Silencing the synaptic output of L1 strongly suppressed voltage changes of LPTCs (Joesch et al., 2010) as well as behavioral responses of the fly (Clark et al., 2011) to the horizontal motion of light edges. In contrast, suppression of neuronal and behavioral responses to horizontal motion of dark edges was observed when the same genetic manipulation was done with L2. However, where exactly in these two pathways this half-wave rectification is implemented is controversial. The authors of one study concluded that L2 itself already provides a half-wave rectified output signal, because prominent calcium signals of L2 terminals were observed only in response to off-stimuli (Reiff et al., 2010). In contrast, in another study L2 as well as L1 were found to respond to on- and off-stimuli with decreases and increases in cytosolic calcium, respectively, in their synaptic terminals (Clark et al., 2011). Half-wave rectification was therefore argued to be implemented downstream of L1 and L2. Further support for this view was the observation that flies, in which the output of either L1 or L2 was silenced, still showed behavioral responses to reverse-phi motion. This type of illusory motion percept is elicited by a brightness change at one location followed by a brightness change of opposite polarity at a neighboring location, and therefore requires on- and off-channels to interact (Clark et al., 2011). Thus it is still not clear how information about different polarities of brightness changes is further processed and how lateral interaction between optical cartridges is involved in motion and pattern detection. Electrophysiological recordings from so-called “full-wave rectifying transient cells” in the medulla of *Calliphora* suggest that these cells pool on- and off-inputs, which adapt individually to repeated stimulation, but the spatial layout of these input elements was not determined (Wiederman et al., 2008). Recent anatomical work suggests that L4 serves as a lateral connection that collects signals from the L2-neurons of the adjacent lamina column, providing input to the medulla TM2 neuron (Takemura et al., 2011). This input organization could explain the spatial offset between on- and off- responses.

The retinotopic activation of the tangential structures we found in the medulla suggests that the arborizations of these cells are not simply output regions that distribute signals from one column to its neighbors, but serve as input structures that integrate signals from a larger population. A correlation of orientation tuning to dendritic morphology has been shown to exist for the small columnar T2 neurons in the medulla (Douglass and Strausfeld, 2003), and the same principle might also account for the spatial filtering that leads to orientation selectivity in larger medulla tangential structures (Srinivasan and Dvorak, 1980). This extraction of specific orientation information prior to motion computation might be functionally beneficial to reduce noise introduced by motion which does not originate from coherent structures of a certain extent in the environment. A similar design principle appears to exist in vertebrate visual cortex. Here, motion-processing areas of the extrastriate cortex receive their main input from the primary visual cortex, in which orientation selectivity is an essential neuronal characteristic (Orban, 2008).

Both the correlation of spatially separated on- and off channels and the use of dendritic morphological layout for spatial filtering are established concepts in vertebrate vision (Hirsch and Martinez, 2006; Vaney et al., 2012). In the general approach to develop models for visual feature extraction, further investigation of the similarities and differences between taxa (Sanes and Zipursky, 2010; Borst and Euler, 2011) will remain a challenging and rewarding field of study.

### Conflict of interest statement

The authors declare that the research was conducted in the absence of any commercial or financial relationships that could be construed as a potential conflict of interest.

## Abbreviations

DSI: direction selectivity index
LPTC: lobula plate tangential cell
OSI: orientation selectivity index
ROI: region of interest.
